# Two new polypore species *Coriolopsis albescens* and *Polyporus laoshanensis* (*Basidiomycota*, *Polyporales*) from Jiangsu Province, China

**DOI:** 10.3897/mycokeys.139.209057

**Published:** 2026-09-04

**Authors:** Qiu-Yue Zhang, Yu-Han Cao, Xing-Xu Chen, Mo-Han Huang, Zhuo Wang, Feng-Ming Shi, Shu Lv, Hai-Dong Li

**Affiliations:** 1 College of Forestry, Nanjing Forestry University, Nanjing, Jiangsu 210037, China College of Forestry, Nanjing Forestry University Nanjing China; 2 Key Laboratory of State Forestry and Grasland Administration on Wildlife Evidence Technology, Nanjing Police University, Naning, Jiangsu 210023, China Key Laboratory of State Forestry and Grasland Administration on Wildlife Evidence Technology, Nanjing Police University Naning China; 3 Nanjing Laoshan Forest Biodiversity Observation Station, Jiangsu 210037, China Nanjing Laoshan Forest Biodiversity Observation Station Jiangsu China

**Keywords:** New taxa, phylogenetic analyses, *
Polyporaceae
*, taxonomy, white rot

## Abstract

Based on a systematic survey of polypores in Jiangsu Province, China, two previously undescribed species of *Polyporaceae* were discovered. Through a combination of morphological examination and phylogenetic analyses, these specimens were identified as new to science: *Coriolopsis albescens***sp. nov**. and *Polyporus laoshanensis***sp. nov**. *Coriolopsis albescens* is characterized by its annual, resupinate basidiomata with a white to pale brown pore surface, round to angular pores of 0.5–1.5 per mm, a trimitic hyphal system, and cylindrical basidiospores measuring 8–12 × 3.2–4 µm. *Polyporus laoshanensis* is distinguished by its annual, solitary, elliptical basidiomata, obvious black stipe, pale yellow pileal surface with dark brown margins, glabrous, occasionally with distinct concentric zones and radial stripes, angular pores (3–6 per mm), a dimitic hyphal system, and cylindrical basidiospores measuring 8–12 × 3–5 μm. Illustrated descriptions of both novel taxa are provided. This work contributes to the documentation of *Polyporaceae* diversity and the taxonomy of wood-decaying fungi in China.

## Introduction

The *Polyporaceae* Fr. ex Corda (*Basidiomycota*, *Polyporales*) is a diverse family of fungi with a worldwide distribution (Wu et al. 2022; Zhao et al. 2024, 2026). Most species of *Polyporaceae* possess a poroid hymenophore, while corticioid and hydnoid hymenophores also occur in some members of this family (Dai et al. 2004; Yuan et al. 2026). These fungi are fundamental agents of wood decomposition in forest ecosystems worldwide (Cui et al. 2019; Hyde et al. 2024). The decay process enables the enzymatic degradation of major wood components such as lignin, cellulose, and hemicellulose, and plays an essential role in material decomposition and nutrient cycling in forest ecosystems (Targino de Oliveira et al. 2024; Zhao et al. 2025). Some species of polypores have significant economic values because of their pathogenetic ecology and, sometimes, supposed medicinal properties (Dai et al. 2000; Vainio et al. 2011; Wu et al. 2019; Yuan et al. 2023; Bhunjun et al. 2024; Ghobad-Nejhad et al. 2024; Cui et al. 2025). Although *Polyporaceae* has a long history of study, and many species have been described from China (Dai et al. 2021; Yuan et al. 2021; Mao et al. 2023; Wang et al. 2025), numerous undescribed species still remain to be uncovered within this fungal family.

The genus *Coriolopsis* Murrill belongs to *Polyporaceae* and is defined by morphological features and molecular phylogenetic evidence (Corner 1989; Justo and Hibbett 2011). It is characterized by basidiomata that are typically ochraceous to brown, with a trimitic hyphal system, and cyanophilous skeletal hyphae that typically exhibit an ochraceous coloration (Murrill 1905; Tomsovský et al. 2006; Cui et al. 2019). Historically, the taxonomic delineation of this genus has been complex and subject to revision (Tomsovský et al. 2006). There are more than 80 records of *Coriolopsis* in Index Fungorum, and most of these species have been synonymized and transferred due to morphological similarities with closely related genera, such as *Funalia* Pat., *Lenzites* Fr., *Pycnoporus* Karst. and *Trametes*. At present, only about 20 species of *Coriolopsis* are currently accepted in Index Fungorum, yet molecular sequence data are available for merely four of these accepted species (Jahn 1963; Tomsovský et al. 2006; Cui et al. 2019). Recently, the advent of molecular phylogenetics has been instrumental in clarifying the relationships within this group. The current classification, which integrates morphology with molecular data, reflects a more natural system, resulting in the re-evaluation or transfer of many species (Li et al. 2016; Cui et al. 2019; Lücking et al. 2020; Targino de Oliveira et al. 2024). *Coriolopsis* is polyphyletic within *Polyporaceae*, causing its taxonomic position to remain in doubt (Justo and Hibbett 2011). Despite this, based on molecular phylogenetics, Tomsovský et al. (2006) and Cui et al. (2019) demonstrated that most *Coriolopsis* species form a well-supported clade distinct from the main *Trametes* clade. Therefore, precise circumscription of *Coriolopsis* still need to be refined in future research.

*Polyporus* P. Micheli ex Adans. represents one of the oldest and most well-studied groups of polypores, characterized by stipitate basidiomata and diverse morphological features (Ji et al. 2022; Zheng et al. 2024). The genus is widely distributed, inhabiting various forest ecosystems. Morphologically, *Polyporus* species exhibit morphological differences, such as the form of the basidiomata, pileal surface, pore size, stipe characteristics, and basidiospore morphology, which are key diagnostic features for species identification (Sotome et al. 2008; Zhuang and Luo 2025; Luo et al. 2026). The genus is highly heterogeneous and has been traditionally divided into six main morphological groups, namely, the *Polyporus* group, the *Favolus* group (= *Favolus* Fr.), the *Melanopus* group (= *Melanopus* Pat.), the *Polyporellus* group (= *Polyporellus* Karst.), the *Admirabilis* group, and the *Dendropolyporus* group (= *Dendropolyporus* (Pouz.) Jülich) (Krüger et al. 2006; Sotome et al. 2008, 2011). However, phylogenetic studies have revealed that *Polyporus* is polyphyletic, with its members distributed across several well-supported clades that do not fully correspond to these traditional morphological classifications (Sotome et al. 2008; Zhou et al. 2016). Recent studies have been undertaken using morphological examination and multi-locus phylogenetic analyses to reconstruct a comprehensive phylogeny within a broadly defined *Polyporus* (Ji et al. 2022; Zheng et al. 2024; Zhuang and Luo 2025; Luo et al. 2026).

During a survey of polypores in Jiangsu Province, China, two undescribed species of *Polyporaceae* were discovered. Based on integrated morphological and phylogenetic analyses, these species were identified as new to science, belonging to the genera *Coriolopsis* and *Polyporus*. This paper provides their formal descriptions and illustrations.

## Materials and methods

### Morphological studies

The specimens were collected from Laoshan National Forest Park, Jiangsu Province in eastern China. The basidiomata were photographed *in situ*, macro-morphology and collection details were noted, and they were taken to the Forest Pathology Laboratory at Nanjing Forestry University (NJFC) in plastic collection boxes. They were then dried at 35 °C for 24 hours. Macromorphological characteristics were investigated based on field notes and color photos of basidiomata. The color codes used were those of Petersen (1996). Micromorphological observations of reproductive structures were conducted using a Nikon Eclipse 80i microscope (Tokyo, Japan). A Nikon Digital Sight DS-L3 camera was used to photograph microscopic structures. Drawings were made with the aid of a drawing tube. Microscopic features, measurements, and drawings were made from slide preparations stained with Cotton Blue and Melzer’s reagent. Basidiospores were measured from sections cut from the hymenophore. To present the variation of basidiospore size, 5% of measurements were excluded from each end of the range and are given in parentheses. The following abbreviations are used: IKI = Melzer’s Reagent, IKI–, KOH = 5% potassium hydroxide, CB = Cotton Blue, CB+ = cyanophilous, CB– = acyanophilous, L = arithmetic average of spore length, W = arithmetic average of spore width, Q = L/W ratios, and n = number of basidiospores/measured from given number of specimens.

### DNA extraction and sequencing

Acetyl trimethylammonium bromide (CTAB) rapid plant genome extraction kit (Aidlab Biotechnologies, Co., Ltd., Beijing, China) was used to extract DNA. The nuclear ribosomal internal transcribed spacer (ITS) region, and nuclear ribosomal large subunit (nLSU) gene were amplified using the selected primer pairs ITS5/ITS4 (White et al. 1990) and LR0R/LR5 (Vilgalys and Hester 1990). The PCR cycling schedule for ITS included an initial denaturation at 95 °C for 3 min or 94 °C for 2 min, followed by 35 cycles at 94 °C for 40 s, 54 °C for 45 s and 72 °C for 1 min, and a final extension of 72 °C for 10 min. The PCR cycling schedule for nLSU included an initial denaturation at 94 °C for 1 min, followed by 35 cycles at 94 °C for 30 s, 50 °C for 1 min and 72 °C for 1.5 min, and a final extension of 72 °C for 10 min. All newly generated sequences in this study were deposited in GenBank and are listed in bold in Table 1.

**Table 1.** Specimens and GenBank accession numbers for the sequences used in this study. Newly generated sequences are in bold. ^T^ indicates type specimens.

**Species**	**Voucher**	**Country**	**ITS**	**nLSU**
*Cerrena caperata*	LE(BIN)-0677	Cuba	AB158316	—
*Cerrena fulvocinerea*	JV1207 5.1 JC	USA	OR911809	OR911803
***Coriolopsis albescens***	**QYZhang 69**	**China/Jiangsu**	**PX625768**	**PX625773**
***Coriolopsis albescens***	**QYZhang 55**	**China/Jiangsu**	**PX625767**	**PX625772**
***Coriolopsis albescens***	**QYZhang 378^T^**	**China/Jiangsu**	**PX625769**	**PX625774**
*Coriolopsis brunneoleuca*	Dai 12087	China	KC867416	KC867435
*Coriolopsis brunneoleuca*	Dai 12118	China	KC867418	KC867436
*Coriolopsis dendriformis*	Cui 6719^T^	—	KC867408	KC867445
*Coriolopsis dendriformis*	Yuan6316	—	KC867409	KC867446
*Coriolopsis gallica*	MOGU 086-19	Italy	OM422731	—
*Coriolopsis gallica*	CBS:547.50	France	MH856754	—
*Cubamyces flavidus*	OAB0047	Benin	MK736966	MK736946
*Cubamyces flavidus*	OAB0090	Benin	MK736967	—
*Cubamyces flavidus*	CBS 158.35	—	MH855616	MH867126
*Cubamyces lactineus*	DMC346	Cameroon	KC589126	KC589152
*Cubamyces lactineus*	CBS 109427	Taiwan	MH862825	—
*Cubamyces lactineus*	OAB0232	Benin	MK736983	MK736948
*Cubamyces lactineus*	BCC 33266	Thailand	GQ982888	GQ982881
*Cubamyces menziesii*	BRFM<FRA>:1368	Martinique	JN645103	—
*Cubamyces menziesii*	Dai6782	—	KC848289	KC848374
*Cubamyces menziesii*	FPRI101	Philippines	JN164933	—
*Cubamyces menziesii*	CBS:453.76	India	MH860991	MH872762
*Datroniella scutellata*	RLG9584T	USA	JN165004	JN164792
*Dichomitus squalens*	Cui 9639	China	JQ780407	JQ780426
*Earliella scabrosa*	PR1209	Puerto Rico	JN165009	JN164793
*Favolus acervatus*	Cui 11053	China	KU189774	KU189805
*Favolus acervatus*	Dai 10749b	China	KX548953	KX548979
*Favolus gracilisporus*	Cui 4292	China	KX548970	KX548992
*Favolus gracilisporus*	Li 1938	China	KX548971	KX548993
*Favolus grammocephalus*	LAH02926	Pakistan: Punjab	PZ429299	PZ429306
*Fomes fasciatus*	52FA_PSRB	USA	JX126905	—
*Fomes fomentarius*	BRNM 840314	Slovakia	OQ474932	—
*Fomes inzengae*	BRNM 840289	Czech Republic	OQ474920	—
*Fomitella supina*	JV0610	Guatemala	KF274645	KF274646
*Fomitella supina*	Nunez 1183	Panama	KF274644	—
*Fomitella supina*	Ryvarden 39027	Puerto Rico	KF274643	—
*Funalia aspera*	NP170-8	Thailand	MK589269	—
*Funalia aspera*	Cui 6725	China	KC867356	KC867477
*Funalia cystidiata*	Dai 12093^T^	China	NR169652	NG075185
*Funalia cystidiata*	Cui 8396	China	KC867392	KC867455
*Funalia floccosa*	Dai 10738	China	KC867377	KC867450
*Funalia floccosa*	O-F-308196^T^	Indonesia	OR892706	OR896863
*Funalia rigida*	3213	Brazil	ON847376	—
*Funalia rigida*	VRTOB09 (URM 93661)	Brazil	OQ702341	OR333970
*Funalia rigida*	VRTO62 (URM 95589)	Brazil	OR892710	OR896866
*Funalia sanguinaria*	Cui 5296	China	KC867388	KC867465
*Funalia sanguinaria*	Dai 9314	China	KC867390	KC867467
*Funalia subgallica*	Cui 6317	China	KC867384	KC867460
*Funalia subgallica*	Dai 6329^T^	China	KC867386	KC867462
*Hexagonia glabra*	Dai 10691	China	JX569733	JX569750
*Hexagonia tenuis*	Cui 8468	China	JX559277	JX559302
*Jorgewrightia guangdongensis*	Cui 13986^T^	China	MG847208	MG847217
*Lentinus longiporus*	DAOM:229479	Canada	AB478880	LC052217
*Lentinus longiporus*	WD2579	Japan	AB478879	LC052218
*Lentinus substrictus*	Wei 1582	China	KU189767	KU189798
*Lentinus substrictus*	Wei 1600	China	KC572022	KC572059
*Lenzites betulinus*	HHB9942sp	USA	JN164983	JN164794
*Lenzites betulinus*	Dai6847	—	KC848305	KC848390
*Mariorajchenbergia subcavernulosa*	Cui 14247	China	MG847213	MG847222
*Megasporia variabilicolor*	URM 88368^T^	Brazil	KX584448	KX619574
*Megasporoporia setulosa*	URM 85679	Brazil	KX584459	OL684780
*Megasporoporia setulosa*	LR 9907	Tanzania	OL678508	OL684780
*Microporus affinis*	Cui 7714	China	JX569739	JX569746
*Microporus flabelliformis*	Dai 11574	China	JX569740	JX569747
*Neodatronia gaoligongensis*	Cui 8055	China	JX559269	JX559286
*Neodatronia sinensis*	Dai 11921^T^	China	JX559272	JX559283
*Neofavolus alveolaris*	MOGU 096-19	Italy	OM530253	—
*Neofavolus cremeoalbidus*	Cui 12412	China	KX899982	KX900109
*Neofavolus cremeoalbidus*	TUMH:50009^T^	Japan	AB735980	AB735957
*Neofavolus mikawai*	Cui 11152	China	KU189773	KU189804
*Neofavolus mikawai*	Dai 12361	China	KX548975	KX548997
*Neofomitella polyzonata*	Dai 10419^T^	China	JX569738	JX569745
*Neofomitella rhodophaea*	TFRI 414	—	EU232216	EU232300
*Picipes ailaoshanensis*	Cui 12585	China	KX900068	KX900183
*Picipes ailaoshanensis*	Cui 12578^T^	China	KX900067	KX900182
*Picipes americanus*	JV 0809-104	USA	KC572003	KC572042
*Picipes americanus*	JV 0509-149^T^	USA	KC572002	KC572041
*Picipes annularius*	Cui 10123^T^	China	KX900060	KX900176
*Picipes atratus*	Dai 13375	China	KX900042	KX900158
*Picipes atratus*	Cui 11289^T^	China	KX900043	KX900159
*Picipes auriculatus*	Yuan 4221	China	KX900064	KX900180
*Picipes auriculatus*	Cui 13616^T^	China	KX900063	KX900179
*Picipes baishanzuensis*	Cui 11395	China	KU189763	KU189794
*Picipes baishanzuensis*	Dai 13418^T^	China	KU189762	KU189793
*Picipes brevistipitatus*	Cui 11345	China	KX900074	KX900188
*Picipes brevistipitatus*	Cui 13652^T^	China	KX900075	KX900189
*Picipes conifericola*	Cui 9950	China	KU189783	KU189814
*Picipes conifericola*	Dai 11114^T^	China	JX473244	KC572061
*Picipes fraxinicola*	Dai 2494	China	KC572023	KC572062
*Picipes fraxinicola*	Wei 6025	China	KC572024	KC572063
*Picipes melanopus*	H 6003449	Finland	JQ964422	KC572064
*Picipes melanopus*	MJ 372-93	Czech	KC572026	KC572065
*Picipes nigromarginatus*	Cui 8113^T^	China	KX900062	KX900178
*Picipes rhizophilus*	Dai 11599	China	KC572028	KC572067
*Picipes rhizophilus*	Dai 16082	China	KX851634	KX851687
*Picipes submelanopus*	Dai 13294	China	KU189770	KU189801
*Picipes submelanopus*	Dai 13296	China	KU189771	KU189802
*Picipes subtropicus*	Li 1928	China	KU189758	KU189790
*Picipes subtropicus*	Cui 2662^T^	China	KU189759	KU189791
*Picipes subtubaeformis*	Cui 10793	China	KU189753	KU189785
*Picipes subtubaeformis*	Dai 11870^T^	China	KU189752	KU189784
*Picipes taibaiensis*	Dai 5741	China	JX489169	KC572071
*Picipes taibaiensis*	Dai 5746^T^	China	KX196783	KX196784
*Picipes tibeticus*	Cui 12225	China	KU189756	KU189788
*Picipes tibeticus*	Cui 12215^T^	China	KU189755	KU189787
*Picipes tubaeformis*	Niemela 6855	Finland	KC572036	KC572073
*Picipes tubaeformis*	JV 0309-1	USA	KC572034	KC572072
*Picipes ulleungus*	Cui 12410	China	KX900022	KX900142
*Picipes virgatus*	CulTENN11219	Argentina	AF516581	AJ488122
*Picipes virgatus*	CulTENN11406	Argentina	AF516582	AJ488122
*Picipes wuyishanensis*	Dai 7409^T^	China	KX900061	KX900177
*Podofomes mollis*	RLG6304sp	USA	JN165002	JN164791
*Podofomes stereoides*	Holonen	Finland	KC415179	KC415196
*Polyporus austrosinensis*	Cui 11140	China	KX900046	KX900162
*Polyporus austrosinensis*	Cui 11126^T^	China	KX900045	KX900161
*Polyporus guianensis*	TENN 58404	Venezuela	AF516566	AJ487948
*Polyporus guianensis*	TENN 59093	Argentina	AF516564	AJ487947
*Polyporus hemicapnodes*	Cui 11259	China	KX851625	KX851679
*Polyporus hemicapnodes*	Dai 13403	China	KX851627	KX851681
*Polyporus lamelliporus*	Dai 12327	China	KX851622	KX851676
*Polyporus lamelliporus*	Dai 15106^T^	China	KX851623	KX851677
***Polyporus laoshanensis***	**QYZhang 533** ^T^	**China**	**PZ735506**	**PZ735508**
***Polyporus laoshanensis***	**QYZhang 534**	**China**	**PZ735506**	**PZ735509**
*Polyporus mangshanensis*	Dai 15151^T^	China	KX851796	KX851797
*Polyporus minutissimus*	Wu 970	China	PP035829	PP035826
*Polyporus minutissimus*	Wu 971^T^	China	PP035830	PP035827
*Polyporus miscanthi*	Dai 24655	China	PP407553	PP407555
*Polyporus miscanthi*	Dai 24656	China	PP407554	PP407556
*Polyporus parvovarius*	Yuan 6639	China	KX900049	KX900165
*Polyporus parvovarius*	Dai 13948	China	KX900050	KX900166
*Polyporus radicatus*	DAOM198916	Canada	AF516584	AJ487955
*Polyporus radicatus*	TENN 58831	USA	AF516585	AJ487956
*Polyporus sinosquamosus*	Dai 26113b	China	PQ306796	PQ306793
*Polyporus sinosquamosus*	Dai 29814	China	PQ306797	PQ306794
*Polyporus sinosquamosus*	Dai 29815	China	PQ306798	PQ306795
*Polyporus subvarius*	WD2368	Japan	AB587643	AB587638
*Polyporus subvarius*	Yu 2^T^	China	AB587632	AB587621
*Polyporus varius*	Cui 12249	China	KU507581	KU507583
*Polyporus varius*	Dai 13874	China	KU189777	KU189808
*Pseudofavolus tenuis*	Niemela-9032	Zambia	KY948738	KY948842
*Trametes cingulata*	OAB0114	Benin	MK736971	MK736950
*Trametes cingulata*	OAB0161	Benin	MK736975	MK736951
*Trametes cinnabarina*	Dai 14386	China	KX880629	KX880667
*Trametes cinnabarina*	Cui 7096	—	KC848330	KC848414
*Trametes conchifer*	FP106793sp	USA/Mississippi	JN164924	JN164797
*Trametes conchifer*	FP106793sp	USA	JN164924	JN164797
*Trametes ectypa*	FP103976sp	USA: FLorida	JN164961	—
*Trametes ectypa*	FP106037T	USA	JN164929	JN164803
*Trametes elegans*	PR1133	Puerto Rico	JN164937	—
*Trametes elegans*	FPRI10	Philippines	JN164973	—
*Trametes elegans*	HHB4626sp	USA	JN164950	—
*Trametes elegans*	FP105679sp	USA/Georgia	JN164944	JN164799
*Trametes elegans*	FRI437^T^	—	JN164985	—
*Trametes elegans*	FPRI390	Philippines	JN164921	—
*Trametes gibbosa*	DMC815	Cameroon	KC589144	KC589164
*Trametes gibbosa*	L11664sp	England	JN164943	JN164800
*Trametes glabrorigens*	Cui 13868	China	MK192427	MK192446
*Trametes glabrorigens*	Dai 7894	China	KC867395	—
*Trametes hirsuta*	DMC341	Cameroon	KC589146	KC589166
*Trametes hirsuta*	RLG5133^T^	USA: New York	JN164941	JN164801
*Trametes hostmannii*	BJFC12730	China	KC017755	KC017766
*Trametes junipericola*	145295	—	KC017758	KC017763
*Trametes maxima*	OH189sp	Venezuela	JN164957	JN164804
*Trametes membranacea*	PRSC82	Puerto Rico	JN164945	JN164805
*Trametes neovillosa*	FP103050sp^T^	USA/Florida	JN164958	JN164806
*Trametes ochracea*	HHB13445sp	USA/Michigan	JN164954	JN164812
*Trametes ochracea*	Dai 2005	China	KC848272	KC848357
*Trametes palisotii*	OAB0118	Benin	MK736980	MK736956
*Trametes palisotii*	OAB0198	Benin	MK736982	MK736958
*Trametes parvispora*	OAB0022^T^	Benin	MK736989	MK736964
*Trametes parvispora*	OAB0023	Benin	MK736990	MK736965
*Trametes polyzona*	DMC370	Cameroon	KC589125	KC589151
*Trametes polyzona*	Cui 11040	China	KX880647	KX880689
*Trametes polyzona*	Cui 11040	China	KR605824	KR605767
*Trametes pubescens*	FP101414sp	USA/Wisconsin	JN164963	JN164811
*Trametes punicea*	BCC26408	Thailand	FJ372685	FJ372707
*Trametes punicea*	BCC27595	—	FJ372686	FJ372708
*Trametes socotrana*	BJFC12724	China	KC848313	KC848397
*Trametes socotrana*	OAB0162	Benin	MK736988	MK736963
*Trametes strumosa*	Dai 27320	China	PV191138	PV191239
*Trametes strumosa*	LWZ 20210924-16a	China	ON897965	ON885424
*Trametes suaveolens*	Z2	—	PX966786	PX954113
*Trametes suaveolens*	KMRB17121435	—	ON402823	ON402846
*Trametes trogii*	LG1	Italy	KJ093492	—
*Trametes trogii*	BRFM<FRA>:974	France	JN645099	—
*Trametes trogii*	HMUT 1266	China	OM914752	OM914766
*Trametes trogii*	HMUT 1267	China	OM914753	OM914767
*Trametes trogii*	JV1310	Czech Republic	OR911811	OR911805
*Trametes trogii*	TGC-P-3	China	KR264980	—
*Trametes trogii*	20190810-1b	China	PZ330525	—
*Trametes trogii*	YU0241	China	PV962375	—
*Trametes trogii*	HKAS 145595	China	PV077792	—
*Trametes trogii*	MO546205	USA: Arizona	PQ100183	—
*Trametes trogii*	KMRB15052235	South Korea	ON402826	ON402847
*Trametes trogii*	YG-G14	Uzbekistan	MT534629	MT534627
*Trametes trogii*	LSPQ-NSM-121	Canada	KU761249	
*Trametes versicolor*	FP135156sp	USA	JN164919	JN164809
*Trametes villosa*	CBS 678.70	USA	MH859898	MH871688
*Trametes villosa*	CBS 334.49	USA	MH856545	MH868069
*Yuchengia narymica*	Dai 7050	China	JN048776	JN048795

### Phylogenetic analyses

Phylogenetic trees of *Coriolopsis* and *Polyporus* were constructed using the two concatenated datasets (ITS + nLSU), and phylogenetic analyses were performed with Maximum Likelihood (ML) and Bayesian Inference (BI) methods. *Earliella scabrosa* (Pers.) Gilb. & Ryvarden was used as an outgroup in the phylogenetic analysis of *Coriolopsis* (Li et al. 2016; Targino de Oliveira et al. 2024). *Trametes conchifer* (Schwein.) Pilát, and *T. polyzona* (Pers.) Justo were selected as outgroups in the phylogenetic analyses of *Polyporus* (Zhang et al. 2024). New sequences and reference sequences were aligned separately using MAFFT v.74 (Katoh et al. 2017; http://mafft.cbrc.jp/alignment/server/) with the G-INS-I iterative refinement algorithm and optimised manually in BioEdit v.7.0.5.3 (Hall 1999). The separate alignments were then concatenated using PhyloSuite v.1.2.2 (Zhang et al. 2020).

RAxML 7.2.8 was used to infer ML trees for both datasets with the GTR+I+G model of site substitution, including estimation of Gamma-distributed rate heterogeneity and a proportion of invariant sites (Stamatakis 2006). The branch support was evaluated with a bootstrapping method of 1,000 replicates (Hillis and Bull 1993).

For BI, the best-fit partitioning scheme and substitution model were determined by using ModelFinder (Kalyaanamoorthy et al. 2017) via the “greedy” algorithm, with branch lengths estimated as “linked” and based on the AICc. The BI was conducted with MrBayes 3.2.6 in two independent runs, each of which had four chains for 20 million generations and started from random trees (Ronquist et al. 2012). Trees were sampled every 1,000 generations. The first 25% of the sampled trees were discarded as burn-in and the remaining ones were used to reconstruct a majority rule consensus and calculate Bayesian Posterior Probabilities (BPP) of the clades.

Phylogenetic trees were visualized using FigTree version 1.4.4. Branches that received bootstrap support (BS) for ML and BPPs greater than or equal to 75% (ML) and 0.95 (BPP) were considered significantly supported.

## Results

### Phylogeny

**Figure 1.**
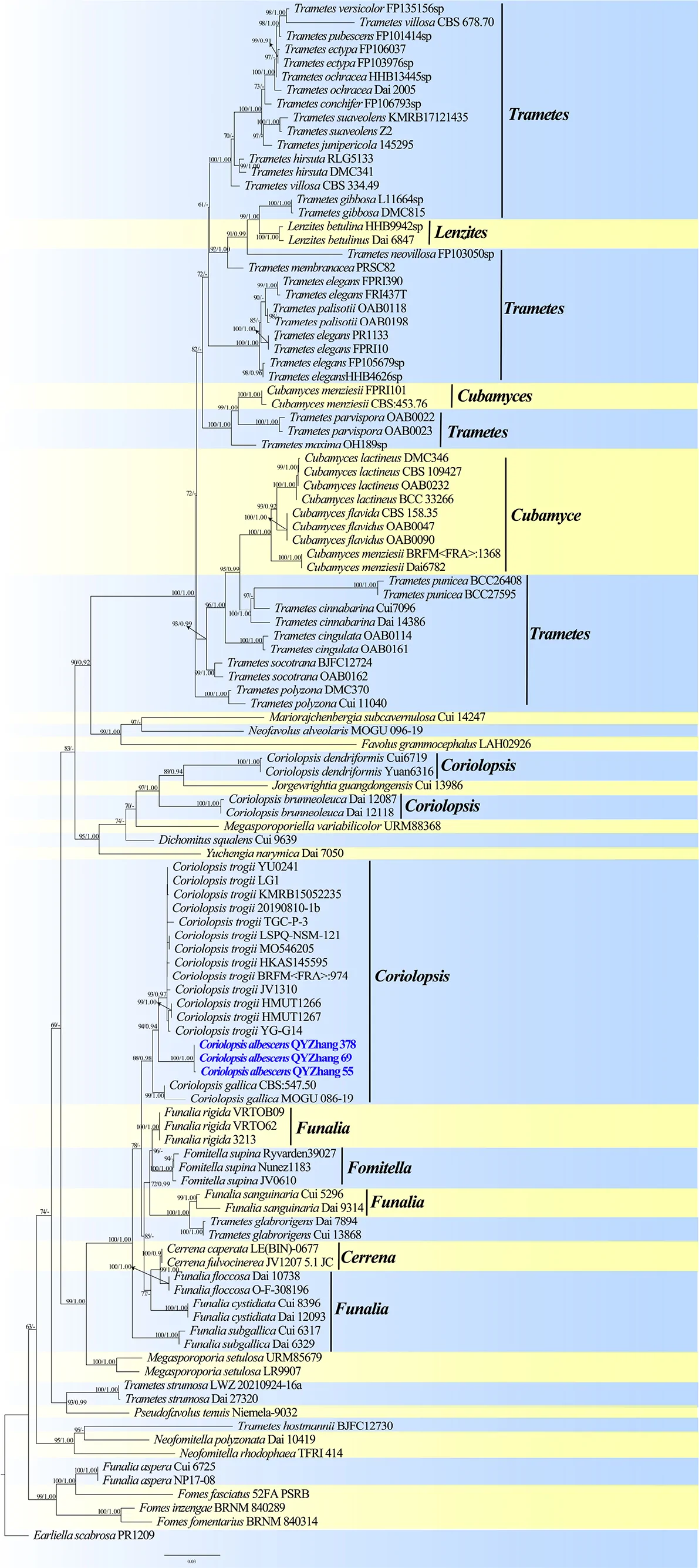
Maximum Likelihood (ML) tree illustrating the phylogeny of *Coriolopsis* and related genera, based on a combined ITS + nLSU dataset. Branches are labelled with parsimony bootstrap values (ML) higher than 50% and Bayesian Posterior Probabilities (BPPs) more than 0.90.

In this study, the combined ITS + nLSU dataset (Fig. 1) included sequences from 113 specimens, representing 58 species of *Coriolopsis* and related genera. The dataset had an aligned length of 2,012 characters including gaps, consisting of 666 characters from ITS and 1,346 characters from nLSU (Table 1). ModelFinder suggested the best models were GTR + F + I + G4 for ITS and nLSU for the Bayesian analysis. The BI analysis resulted in a concordant topology with an average standard deviation of split frequencies of 0.004126. In Fig. 1, the phylogenetic analyses revealed that the new species, *Coriolopsis albescens* (QY Zhang 55, QY Zhang 69, and QY Zhang 378), formed a monophyletic and highly supported clade (100/1.00) that was closely related to *Coriolopsis trogii* Berk. In addition, the genus *Coriolopsis* exhibits considerable genetic divergence within the ITS fragment. Previous taxonomic studies have widely adopted the ITS region for molecular identification of this genus.

**Figure 2.**
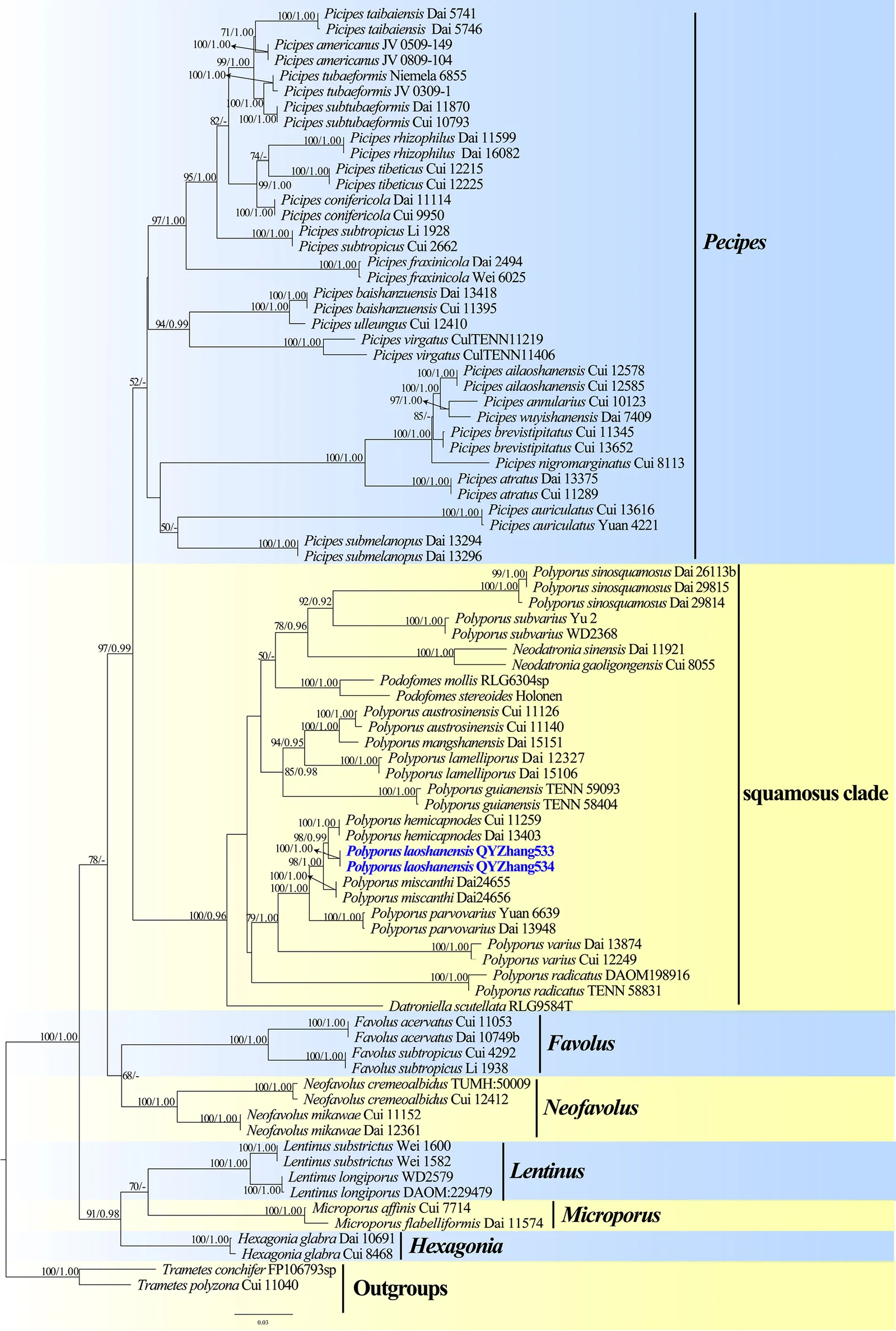
Maximum Likelihood (ML) tree illustrating the phylogeny of *Polyporus* and related genera, based on a combined ITS + nLSU dataset. Branches are labelled with parsimony bootstrap values (ML) higher than 50% and Bayesian Posterior Probabilities (BPPs) more than 0.90.

The combined ITS + nLSU dataset (Fig. 2) included sequences from 87 specimens, representing 48 species of *Polyporus* and related genera. The dataset had an aligned length of 1,799 characters including gaps, consisting of 619 characters from ITS and 1,180 characters from nLSU (Table 1). ModelFinder suggested that the best models were GTR + F + I + G4 for ITS and nLSU for the Bayesian analysis. The BI analysis resulted in a concordant topology with an average standard deviation of split frequencies of 0.003455. In Fig. 2, the phylogram inferred from ITS + nLSU revealed that a new lineage with high support (100/1.00) nests in the squamosus clade in *Polyporus*, herein named *Polyporus laoshanensis*. The new species is closely related to *P. hemicapnodes* Berk. & Broome and *P. miscanthi* Q.Y. Zhang & Y.C. Dai, and *P minutissimus* Q.Y. Zhang, Z.W. Zheng & F. Wu.

In Figs 1, 2, the ML and BI analyses resulted in nearly identical topologies and only the ML tree is presented with the bootstrap supports for ML and the BPP, not less than 50% and 0.90, respectively. In addition, we provide BLAST results in NCBI of the holotype specimen (QY Zhang 378) of *Coriolopsis albescens* and the holotype specimen (QY Zhang 533) of *Polyporus laoshanensis* (Table 2).

**Table 2.** BLAST analysis for the two species in the NCBI nucleotide database.

**Specimens**	**Marker**	**Closest match in GenBank (Species / Isolate Voucher)**	**Accession No**.	**Query Cover (%)**	**Percent Ident (%)**
QYZhang 378	**ITS**	*Coriolopsis trogii*	MN726442	97	97.14
		*Coriolopsis trogii*	MG779615	98	96.86
		*Coriolopsis trogii*	OQ053212	98	96.86
QYZhang 533	**ITS**	*Polyporus brevibasidiosus*	ON553391	99	97.08
		*Polyporus miscanthi*	PP407554	98	97.34
		*Polyporus brevibasidiosus*	PQ804890	97	97.34

### Taxonomy

#### Coriolopsis albescens

Taxon classificationFungiPolyporalesPolyporaceae

Q.Y. Zhang sp. nov.

F8BD1223-D4DE-5D94-8396-0507E353D7F5

862751

Figs 3, 4

##### Diagnosis.

Differs from other *Coriolopsis* species by its resupinate or pulvinate, soft sponge-like basidiomata, and white to pale brown pore surface.

**Figure 3.**
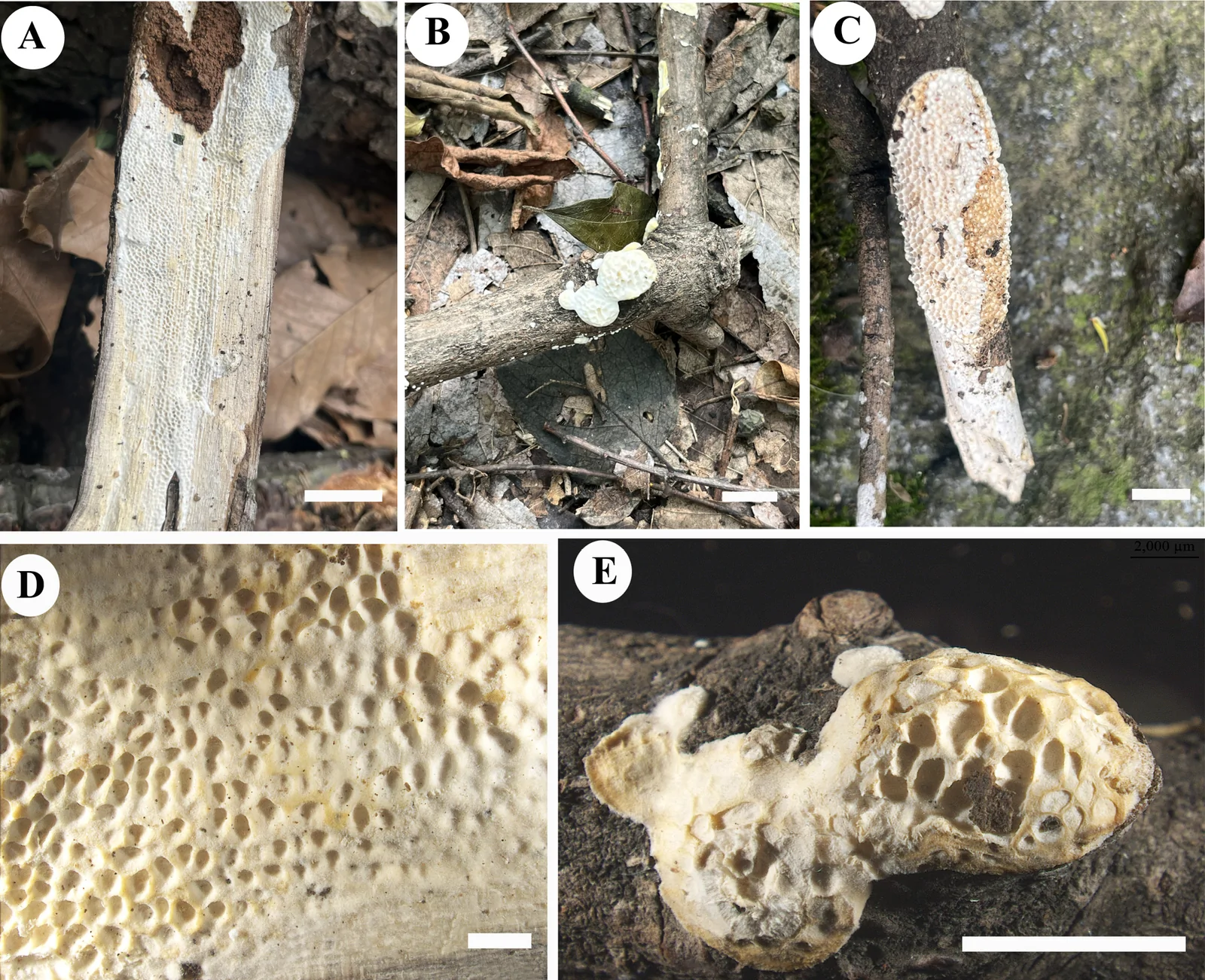
Basidiomata of *Coriolopsis albescens*. **A, D**. QY Zhang 55; **B, E**. QY Zhang 69; **C**. holotype, QY Zhang 378. Scale bars: 1 cm (**A–C, E**); 2 mm (**D**).

**Figure 4.**
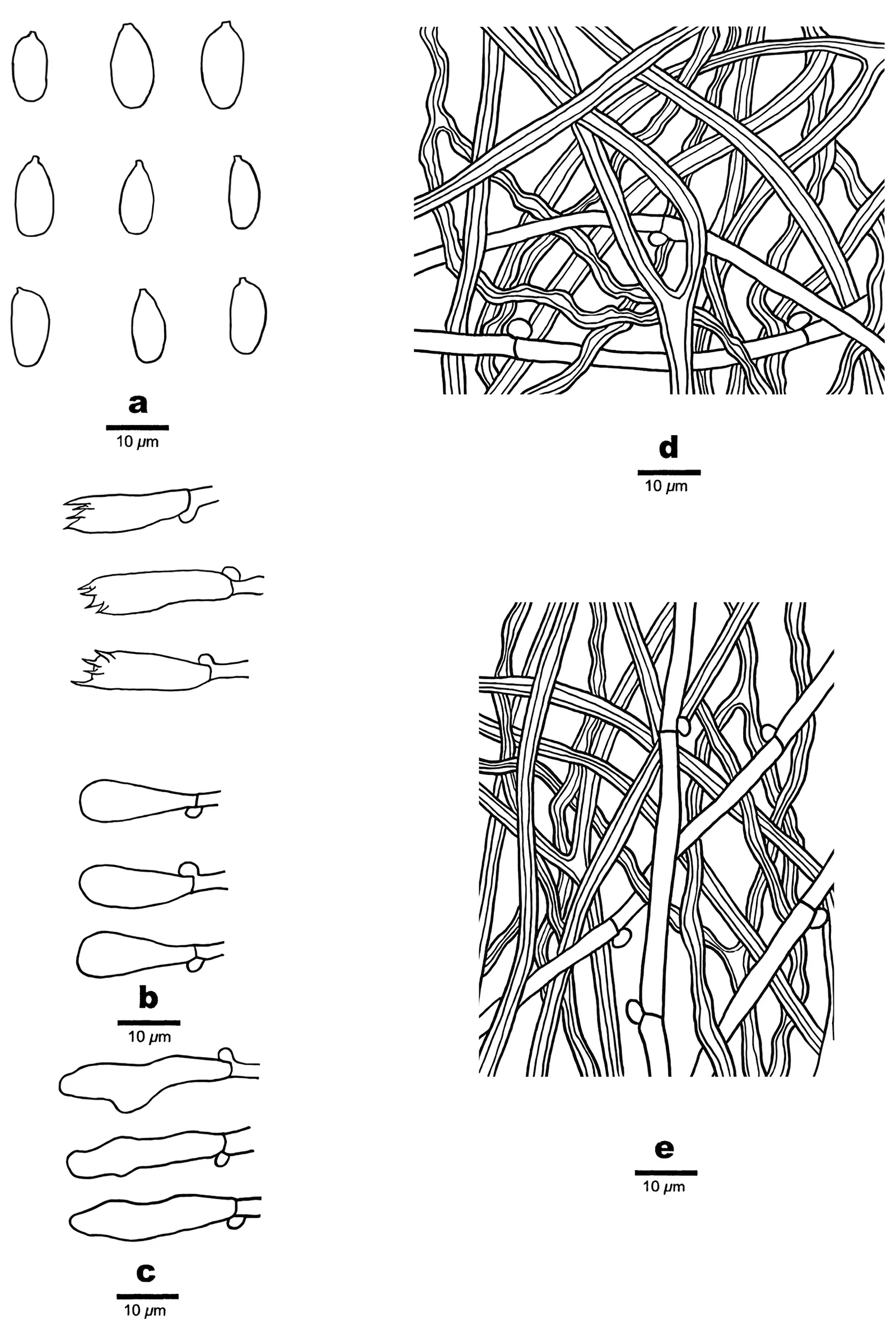
Microscopic structures of *Coriolopsis albescens* (holotype, QYZhang 378). **a**. Basidiospores; **b**. Basidia and basidioles; **c**. Cystidioles; **d**. Hyphae from tube trama; **e**. Hyphae from context.

##### Type.

China • Jiangsu Province, Nanjing, Laoshan National Forest Park, 32°06'N, 118°37'E, on fallen angiosperm branch, 12 September 2025, QY Zhang 378 (holotype, NJFC).

##### Etymology.

*albescens* (Lat.): referring to the basidiomata which are white when juvenile and become pale brown when mature.

##### Description.

***Basidiomata***. Annual, resupinate, without odor or taste when fresh, soft sponge-like texture when dry, up to 12 cm long, 2.5 cm wide and 4 mm thick. Pore surface white to pale brown, glabrous, without concentric zones; pores round to angular, 0.5–2 per mm; dissepiments thin, entire. Context white, sponge-like texture, up to 3 mm thick. Tubes slightly paler than context, soft coriaceous, up to 1 mm long.

***Hyphal structure***. Hyphal system trimitic; generative hyphae bearing clamp connections; skeletal and binding hyphae dominant, thick-walled to subsolid, dextrinoid, CB–; tissues darkening in KOH.

***Context***. Generative hyphae infrequent, hyaline, thin walled, slightly branched, 2.5–3.5 μm in diam; skeletal hyphae dominant, pale yellowish-brown to yellowish brown, thick-walled to subsolid, occasionally branched, interwoven, 2.5–5.5 μm in diam; binding hyphae pale yellowish-brown to yellowish-brown, thick-walled to subsolid, frequently branched, interwoven, 2.5–4.5 μm in diam.

***Tubes***. Generative hyphae infrequent, hyaline, thin walled, frequently branched, 2–2.5 μm in diam; skeletal hyphae dominant, pale yellowish-brown to yellowish brown, thick-walled, occasionally branched, frequently collapsed when dry, interwoven, 2.5–5 μm in diam; binding hyphae pale yellowish-brown to yellowish-brown, thick-walled to almost solid, frequently branched, interwoven, 1.5–4 μm in diam.

***Hymenium***. Cystidia absent; fusoid cystidioles occasionally present, hyaline, thin-walled, 18–35 × 4–6 μm. Basidia clavate, with four sterigmata and a basal clamp connection, 15–30× 5–9.5 μm, L = 23.20 μm, W = 6.37 μm; basidioles similar in shape to basidia, but slightly smaller. Basidiospores mostly oblong-ellipsoid, sometimes with a slight lateral curvature (reniform to somewhat allantoid), with a broadly rounded centre and tapering gradually to a distinct basal apiculus, hyaline, thin-walled, smooth, IKI–, CB–, 8–12(–13) × (3–)3.2–4(–4.5) μm, L = 10.98 μm, W = 3.90 μm, Q = 2.80–2.83 (n = 60/2).

##### Additional specimens examined.

China • Jiangsu Province, Nanjing, Laoshan National Forest Park, 32°06'N 118°37'E, on fallen angiosperm branch, 17 May 2025, QYZhang 55 (NJFC), QYZhang 69 (NJFC).

#### Polyporus laoshanensis

Taxon classificationFungiPolyporalesPolyporaceae

Q.Y. Zhang sp. nov.

FE166CF6-2078-5227-9654-39D88FC26CE6

864711

Figs 5, 6

##### Type.

China • Jiangsu Province, Nanjing, Laoshan National Forest Park, Near the Doushuai temple, 32°06'N 118°37'E, on fallen angiosperm branch, 25 October 2025, QY Zhang 533 (holotype, NJFC).

**Figure 5.**
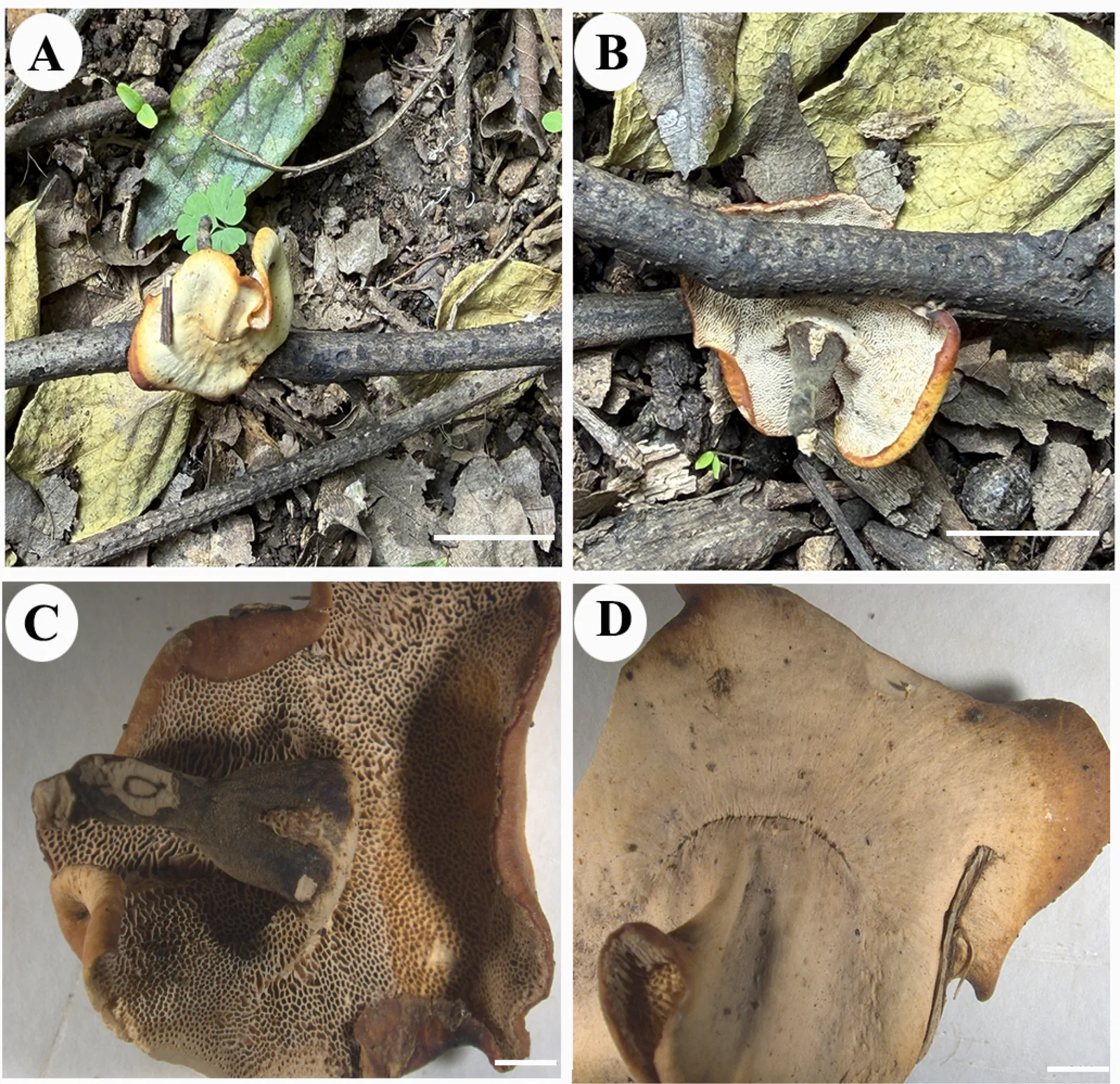
Basidiomata of *Polyporus laoshanensis* (QYZhang 533). Scale bars: 1 cm (**A, B**); 2 mm (**C, D**).

**Figure 6.**
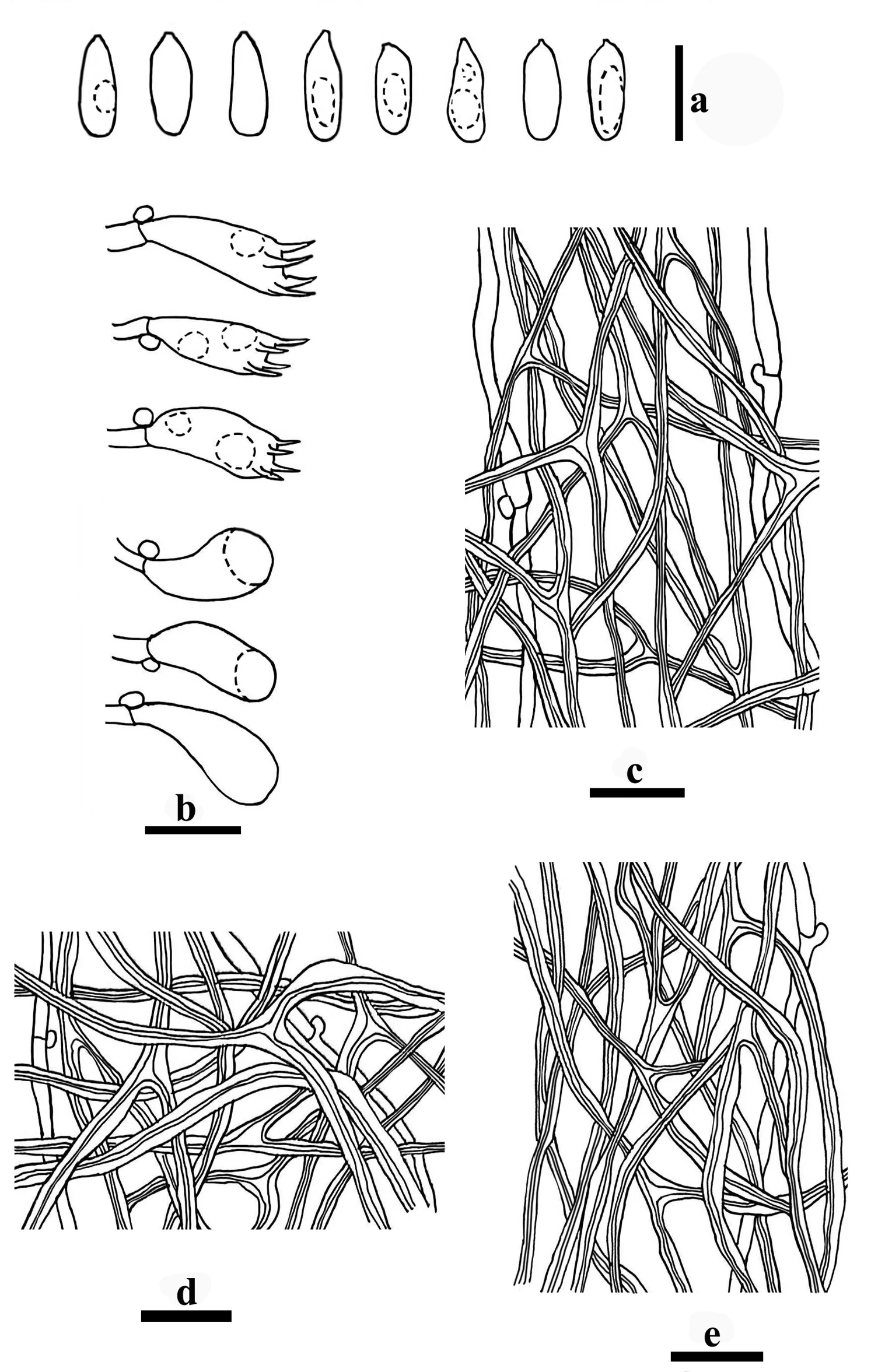
Microscopic structures of *Polyporus laoshanensis* (holotype, QYZhang 533). **a**. Basidiospores; **b**. Basidia and basidioles; **c**. Hyphae from context; **d**. Hyphae from trama; **e**. Hyphae from stipe.

##### Etymology.

*laoshanensis* (Lat.), refers to the locality of the type specimen, Laoshan National Forest Park.

##### Description.

***Basidiomata***. Annual, eccentrically stipitate, solitary, leathery when fresh, soft corky when dry. Pilei irregular, with a depressed center or infundibuliform, up to 1.7 cm in diam and 0.9 mm thick at center. Pileal surface yellowish when fresh, buff when dry, glabrous; margin brown to dark brown, and curved inward. Pore surface white when fresh, cream when dry; pores mostly angular, sometimes sinuous, 3–6 per mm; dissepiments thick, entire. Context cream and soft corky when dry, up to 0.4 mm thick. Tubes paler than pore surface, soft corky when dry, decurrent, up to 0.5 mm long. Stipe black, glabrous, up to 1 cm long and 3 mm in diam.

***Hyphal structure***. Hyphal system dimitic; generative hyphae bearing clamp connections, hyaline, thin-walled; skeleto-binding hyphae thick-walled, with arboriform branches, IKI–, CB+; tissue unchanged in KOH.

***Context***. Generative hyphae infrequent, hyaline, thin-walled, rarely branched, 2–3.5 μm in diam; skeleto-binding hyphae dominant, hyaline, thick-walled with a narrow lumen, frequently branched, strongly interwoven, 1–3 μm diam.

***Tubes***. Generative hyphae infrequent, hyaline, thin-walled, rarely branched, 2–3 μm in diam; skeleto-binding hyphae dominant, hyaline, thick-walled with a narrow lumen, frequently branched, interwoven, 1.5–5 μm in diam. Cystidia and cystidioles absent. Basidia mostly claviform, with four sterigmata and a basal clamp connection, 15–23 × 5–9 μm; basidioles in shape similar to basidia, but slightly smaller.

***Stipe***. Generative hyphae infrequent, hyaline, thin-walled, rarely branched, 2–3 μm in diam; skeleto-binding hyphae dominant, hyaline, thick-walled with a narrow lumen, frequently branched, interwoven, 3–7 μm in diam.

***Basidiospores***. mostly cylindrical tapering to apiculus, hyaline, thin-walled, smooth, sometimes with a guttule, IKI–, CB–, 8–12(–12.5) × 3–5(–5.5) μm, L = 9.49 μm, W = 3.98 μm, Q = 2.32–2.45 (n = 60/2).

##### Additional specimen examined.

China • Jiangsu Province, Nanjing, Laoshan National Forest Park, 32°06'N, 118°37'E, on fallen angiosperm branch, 25 October 2025, QY Zhang 534 (paratype, NJFC).

## Discussion

*Polyporaceae* consists of wood-decay fungi with a global distribution, playing a vital role in nutrient cycling and wood decomposition (Liu et al. 2025; Wang et al. 2025). Recent taxonomic revisions integrating morphological and molecular data have uncovered hidden diversity, resulting in the description of new species and reclassification of genera (Ryvarden and Gilbertson 1993; Jia et al. 2014; Cui et al. 2019; Liu et al. 2025). The combination of morphology with multi-marker phylogenetic inference has proven essential for accurate species delimitation and elucidating evolutionary relationships within this complex family, supporting further biodiversity exploration in poorly studied regions (Wu et al. 2022; Zhao et al. 2024, 2026).

The genus *Coriolopsis* was segregated from *Trametes* as an independent genus (Li et al. 2016; Cui et al. 2019). However, its taxonomic relationships remain highly complex, especially when additional molecular sequences are included in phylogenetic analyses (Olou et al. 2020; Targino de Oliveira et al. 2024). Morphological characteristics overlap extensively between *Coriolopsis* and its related genera, including *Cerrena*, *Funalia*, *Fomitella*, and *Trametes* (Cui et al. 2019; Olou et al. 2020; Yang et al. 2025). Consequently, accurate species delimitation has become increasingly dependent on molecular phylogenetic approaches. In the present study, a new species, *Coriolopsis albescens*, is described based on phylogenetic analyses and morphological characters. Phylogenetically, *Coriolopsis albescens* is closely related to *C. trogii* (*=Trametes trogii*) (Fig. 1). *Coriolopsis trogii* serves as an important model for investigating white rot fungi. Numerous mycologists reported sequences of *C. trogii* collected from Asia, Europe, and the Americas; all these sequences separate from the new species proposed in the present study. However, morphologically, *Coriolopsis trogii* differs from *C. albescens* by having ochraceous buff pore surface and thinner basidiospores (8–12 × 2.54 μm vs. 8–12 × 3.2–4 μm; Ryvarden and Gilbertson 1993). *Coriolopsis albescens* is phylogenetically closely related to *Coriolopsis gallica* (Fr.) Ryvarden (syn. *Trametes gallica*), as they form a joint subclade; however, *C. gallica* differs from *C. albescens* by its thick basidiomata with a hispid to villose, brown to gray pore surface, and longer basidiospores (10–16 μm vs. 8–12 μm in length; Gilbertson and Ryvarden 1986). Furthermore, most species of *Coriolopsis* lack molecular sequence data. Morphologically, the basidiomata of most known congeners range from brown to dark brown. In contrast, *Coriolopsis albescens* has resupinate basidiomata which are white when juvenile and turn pale brown at maturity, showing clear morphological differentiation from its relatives.

The phylogenetic framework of the genus *Polyporus* is well-established and widely accepted, with numerous mycological studies having validated its internal phylogenetic relationships (Ji et al. 2022; Wu et al. 2022; Zhao et al. 2024). Within this genus, the squamosus species have been consistently confirmed as a monophyletic clade in previous multi-locus phylogenetic investigations. However, taxa within the squamosus clade exhibit greatly diverse morphology, making the species within this clade unable to be combined into a monophyletic genus (Cui et al. 2019; Ji et al. 2022; Zhang et al. 2024). Based on the previously established phylogenetic backbone, the present study reconstructed a renewed phylogeny of *Polyporus*. The results robustly place the newly proposed species, *Polyporus laoshanensis*, represented by the specimens QY Zhang533 and 534 within the squamosus clade, and reveal its close phylogenetic affinity with *Polyporus hemicapnodes*, *P. miscanthi*, and *P. parvovarius*. However, *P. hemicapnodes* is readily distinguished from *P. laoshanensis* by smaller pores (6–9 per mm vs. 3–6 per mm) and smaller basidiospores (5.4–7.6 × 2.9–3.8 μm vs. 8–12 × 3–5 μm; Cui et al. 2019). *P. miscanthi* grows on miscanthus, and it can be distinguished from *P. laoshanensis* by its longer stipe (6 cm vs. 2 cm), and sharp, applanate, pale pileus margin, whereas the pileus margin of *P. laoshanensis* is smooth, incurved and dark brown. *P minutissimus* differs from *P. laoshanensis* by its smaller pores (6–9 per mm vs. 3–6 per mm), cream to buff pileal surface, and smaller basidiospores (5–9.2 × 2.2–4 μm vs. 8–12 × 3–5 μm; Zheng et al. 2024). Morphologically, *Polyporus varius* Fr. and *P. laoshanensis* share similar basidiospores and basidia sizes, brown to dark brown stipe; however, *P. varius* has larger basidiomata (up to 8 cm vs. up to 1.7 cm), smaller pores (7–9 per mm vs. 3–6 per mm), and *P. varius* has been reported as widespread in the temperate zone in Europe (north to 70°N), North America, and Asia (Nunez and Ryvarden 1995; Dai 2000; Dai et al. 2007). In addition, *P. americanus* Vlasák & Y.C. Dai resembles *P. laoshanensis* by having a similar buff to honey yellow pore surface and black stipe, but *P. americanus* has a longer stipe (up to 2.7 cm vs. up to 1 cm), the presences of cystidioles, and smaller basidiospores (7–9 × 2.5–3.1 µm vs. 8–12 × 3–5 μm; Dai et al. 2014).

In fact, *Polyporus* is a very complicated genus with more than 3,000 records according to the Index Fungorum, though many species have subsequently been transferred to other genera. Taxonomists have continuously revised and reorganized its taxonomic boundaries in recent years. Studies on *Polyporus* species in China are gradually being carried out, with some species having been described from China by Cui et al. (2019), Ji et al. (2022), Zhang et al. (2024), and Zhuang and Luo (2025), while numerous novel species within this genus have been continuously published. To date, approximately 19 *Polyporus* species have been documented from China.

Wood-rotting fungi, particularly members of the *Polyporaceae*, play an essential role in maintaining ecosystem stability. These fungi decompose wood and promote nutrient cycling, serving as key participants in biosphere material circulation (Si et al. 2011). In recent years, the rapid development and reduced cost of genome sequencing technology have greatly advanced phylogenetic research, effectively promoting taxonomic studies of wood-rotting fungi (Wang et al. 2023; Afshari et al. 2025; Yang et al. 2025). Numerous new species and genera in the *Polyporaceae* have been described so far. Even so, a large number of unknown fungal taxa remain to be discovered (Wu et al. 2022; Yuan et al. 2023; Yang et al. 2025). This study documents two new species of *Polyporaceae* from Nanjing, China. Nanjing lies in the transition zone between subtropical and temperate climates. Its unique biogeography harbours great potential for exploring undiscovered fungal resources. Further large-scale field surveys, combined with morphological observation and molecular phylogenetic analyses, will surely uncover more unrecorded fungal species across China.

## Supplementary Material

XML Treatment for Coriolopsis albescens

XML Treatment for Polyporus laoshanensis

## Additional information

### Artificial Intelligence (AI) use

The authors accept full responsibility for the content of the manuscript, including the disclosure of any use of AI.

No AI tools were used in the preparation of this manuscript.

### Author ORCIDs

Q.-Y. Zhang https://orcid.org/0000-0001-9458-3566

Y.-H. Cao https://orcid.org/0009-0004-5072-3482

H.-D. Li https://orcid.org/0000-0002-6634-9593

### Data availability

All data that support the findings of this study are available in the main text, on GenBank, and in the supplementary materials.

## Supplementary materials

Supplementary material 1 Sequence and peak plot of the type of *Coriolopsis albescens* and *Polyporus laoshanensis* Data type zip https://binary.pensoft.net/file/1764537This dataset is made available under the Open Database License (http://opendatacommons.org/licenses/odbl/1.0/). The Open Database License (ODbL) is a license agreement intended to allow users to freely share, modify, and use this Dataset while maintaining this same freedom for others, provided that the original source and author(s) are credited. Qiu-Yue Zhang, Yu-Han Cao, Xing-Xu Chen, Mo-Han Huang, Zhuo Wang, Feng-Ming Shi, Shu Lv, Hai-Dong Li
