# Supplementary material for: Two new polypore species Coriolopsis albescens and Polyporus laoshanensis (Basidiomycota, Polyporales) from Jiangsu Province, China
Source: MycoKeys. 2026 Sep 4;139:341–62. doi: 10.3897/mycokeys.139.209057 (PMC13570254; doi:10.3897/mycokeys.139.209057)

File: 0001_32026070603043_(378)_[LR0R].ab1 Run Ended: 2026-07-07 19:48:34 Signal G:1235 A:2193 T:3973 C:4031

Sample: 72010524631_32026070603043_(378)_[LR0R]_PW Lane: 4 Base spacing: 14.71 947 bases in 11372 scans

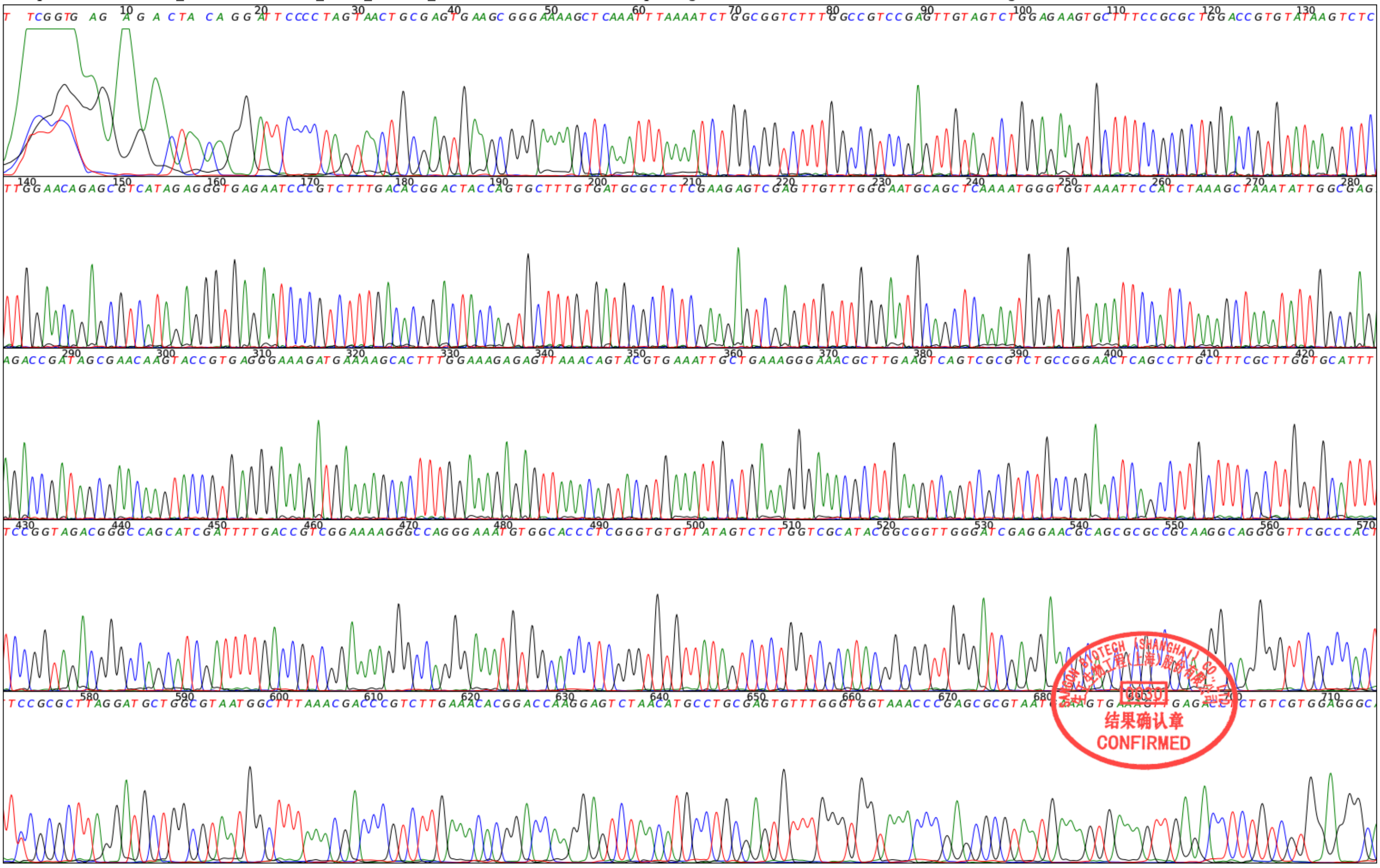

File: 0001_32026070603043_(378)_[LR0R].ab1 Run Ended: 2026-07-07 19:48:34 Signal G:1235 A:2193 T:3973 C:4031

Sample: 72010524631_32026070603043_(378)_[LR0R]_PW Lane: 4 Base spacing: 14.71 947 bases in 11372 scans

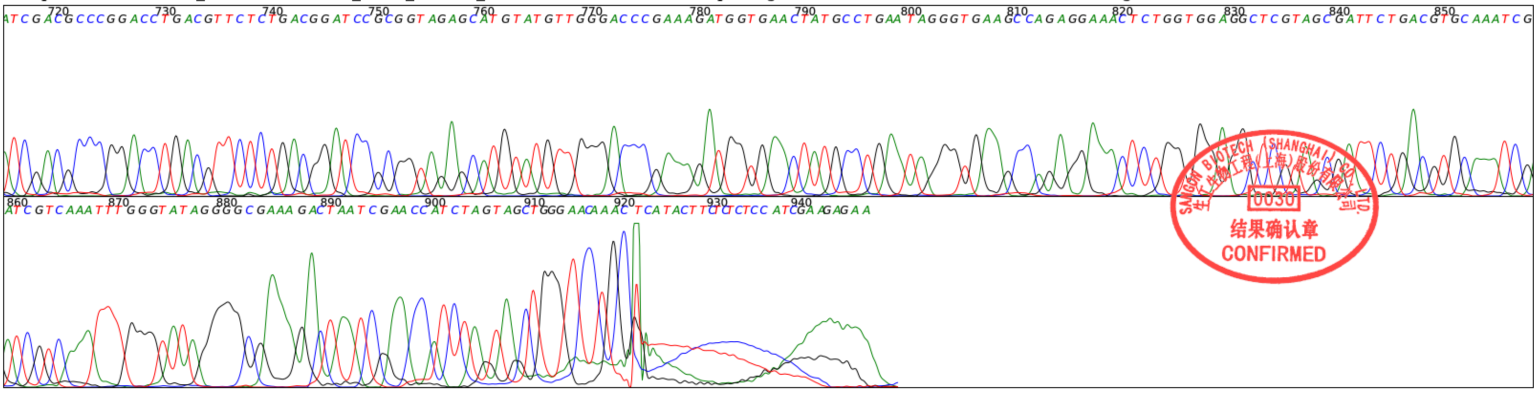

Supplement: Supplementary file 1 [file mycokeys-139-341-s001.zip › 序列/Type of Coriolopsis albescens_QYZhang378/LSU/0001_32026070603043_(378)_[LR0R].pdf]
