# Supplementary material for: Two new polypore species Coriolopsis albescens and Polyporus laoshanensis (Basidiomycota, Polyporales) from Jiangsu Province, China
Source: MycoKeys. 2026 Sep 4;139:341–62. doi: 10.3897/mycokeys.139.209057 (PMC13570254; doi:10.3897/mycokeys.139.209057)

File: 0002_32026070603043_(378)_[LR5].ab1 Run Ended: 2026-07-07 19:48:34 Signal G:1471 A:2588 T:3705 C:4931

Sample: 72010524632_32026070603043_(378)_[LR5]_PW Lane: 4 Base spacing: 14.69 949 bases in 11419 scans Page 1 of 2

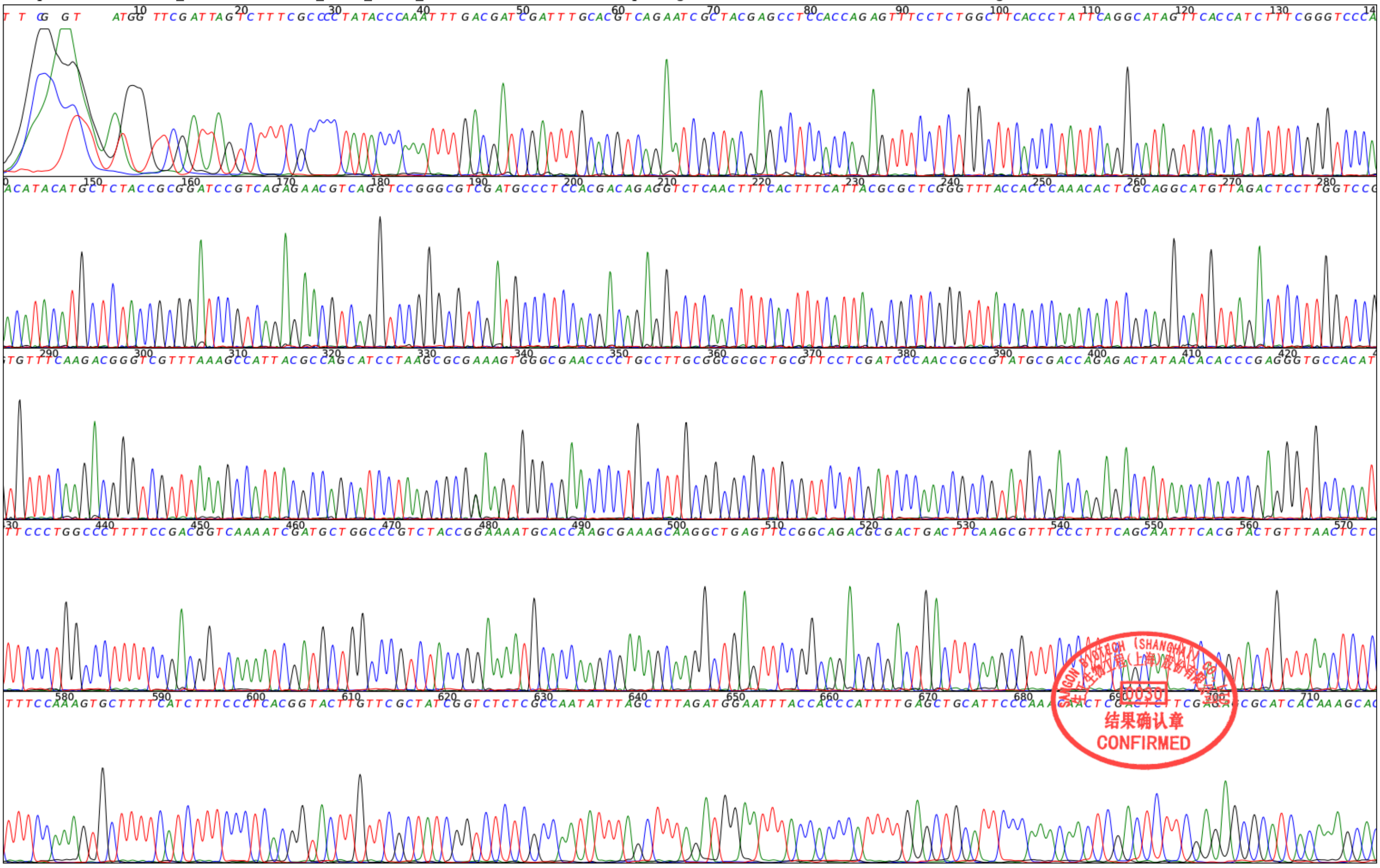

File: 0002_32026070603043_(378)_[LR5].ab1 Run Ended: 2026-07-07 19:48:34 Signal G:1471 A:2588 T:3705 C:4931

Sample: 72010524632_32026070603043_(378)_[LR5]_PW Lane: 4 Base spacing: 14.69 949 bases in 11419 scans

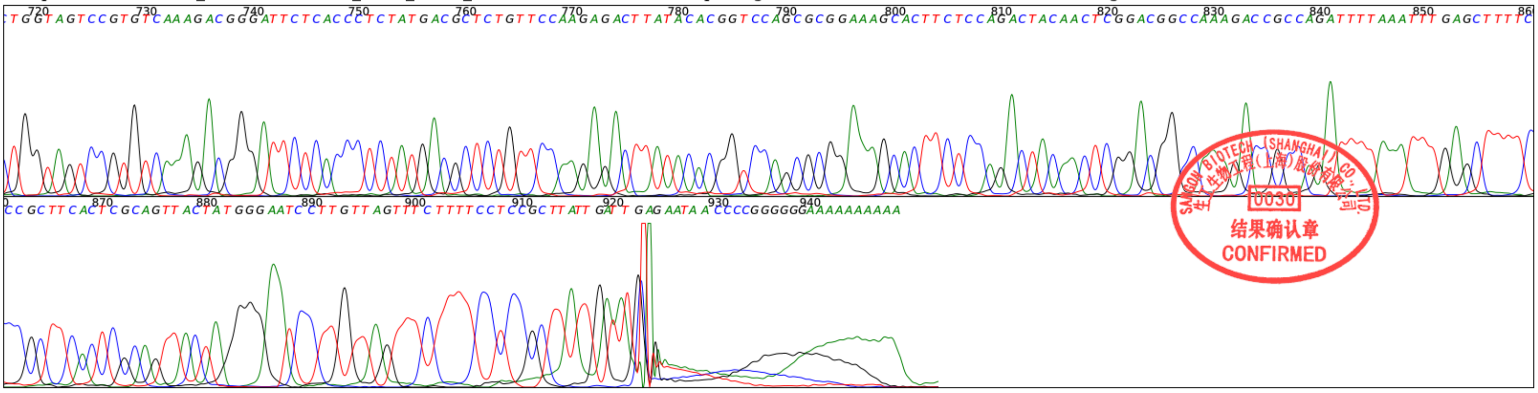

Supplement: Supplementary file 1 [file mycokeys-139-341-s001.zip › 序列/Type of Coriolopsis albescens_QYZhang378/LSU/0002_32026070603043_(378)_[LR5].pdf]
