# Supplementary material for: Two new polypore species Coriolopsis albescens and Polyporus laoshanensis (Basidiomycota, Polyporales) from Jiangsu Province, China
Source: MycoKeys. 2026 Sep 4;139:341–62. doi: 10.3897/mycokeys.139.209057 (PMC13570254; doi:10.3897/mycokeys.139.209057)
Supplement: Supplementary file 1 [file mycokeys-139-341-s001.zip › 序列/Type of Polyporus laoshanensis_QYZhang533/LSU/0013_32026070202233_(533)_[LR0R]_H.pdf]

File: 0013_32026070202233_(533)_[LR0R].ab1   Run Ended: 2026-07-03 06:23:55   Signal G:870 A:1519 T:2593 C:2504

Sample: 72010503275_32026070202233_(533)_[LR0R]_PW   Lane: 4   Base spacing: 14.74   954 bases in 11450 scans

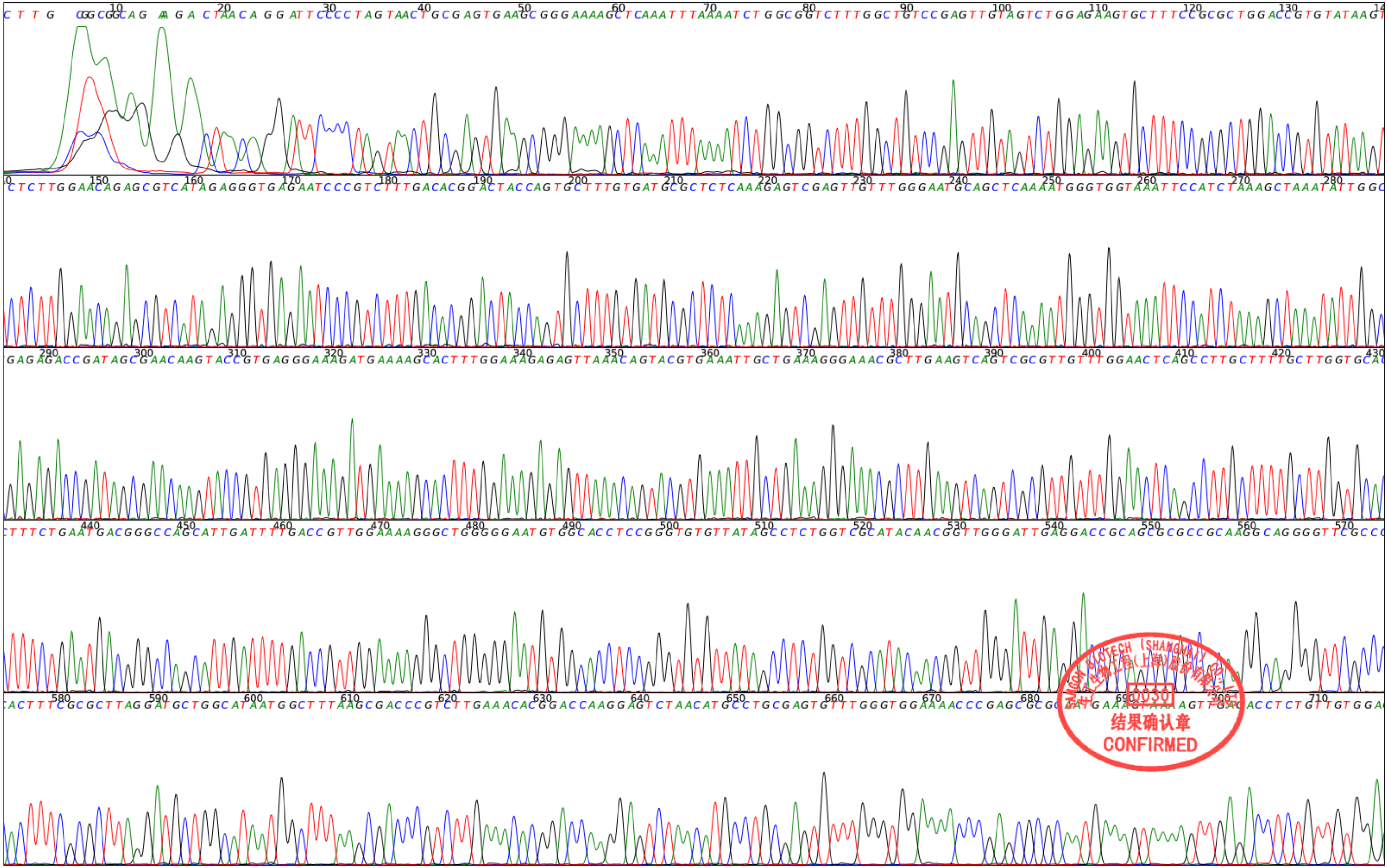

File: 0013_32026070202233_(533)_[LR0R].ab1 Run Ended: 2026-07-03 06:23:55 Signal G:870 A:1519 T:2593 C:2504

Sample: 72010503275_32026070202233_(533)_[LR0R]_PW Lane: 4 Base spacing: 14.74 954 bases in 11450 scans

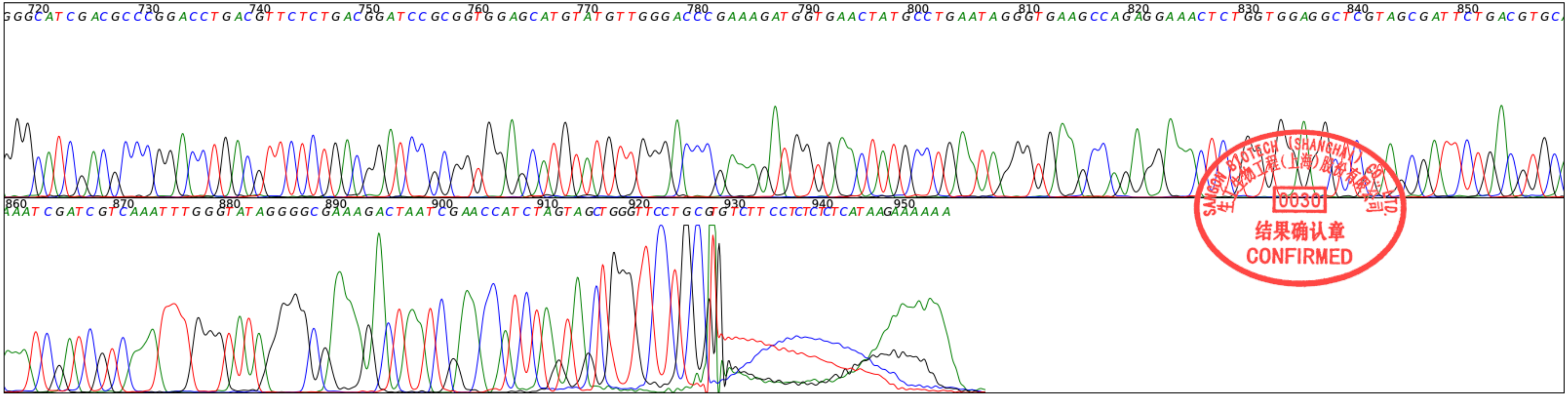

SANGON BIOTECH (SHANGHAI) CO., LTD.
生工生物工程(上海)股份有限公司
0030
结果确认章
CONFIRMED
