# Supplementary material for: Two new polypore species Coriolopsis albescens and Polyporus laoshanensis (Basidiomycota, Polyporales) from Jiangsu Province, China
Source: MycoKeys. 2026 Sep 4;139:341–62. doi: 10.3897/mycokeys.139.209057 (PMC13570254; doi:10.3897/mycokeys.139.209057)

File: 0014_32026070202233_(533)_[LR5].ab1 Run Ended: 2026-07-03 06:23:55 Signal G:616 A:845 T:1364 C:1762

Sample: 72010503276_32026070202233_(533)_[LR5]_PW Lane: 4 Base spacing: 14.71 954 bases in 11457 scans

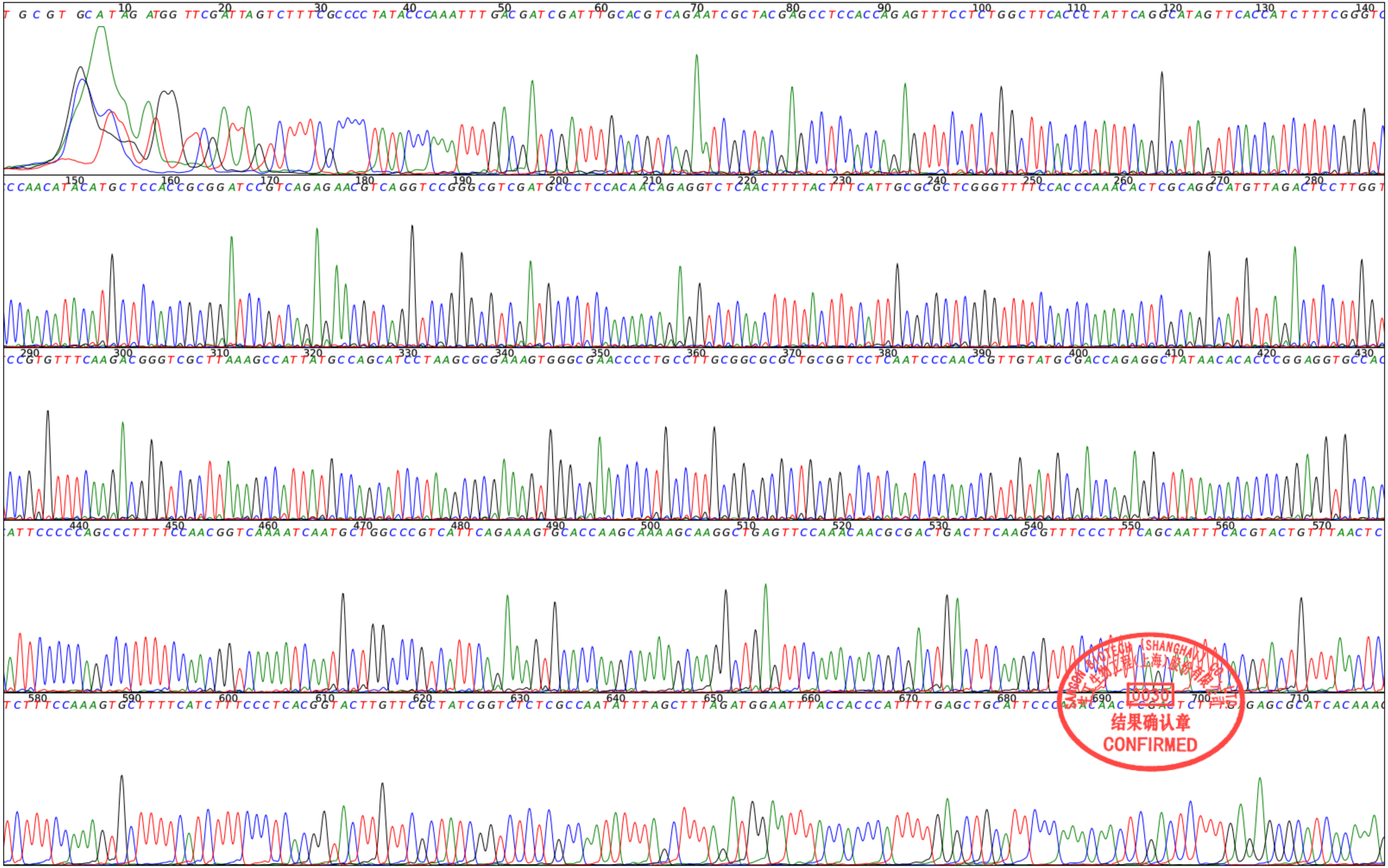

File: 0014_32026070202233_(533)_[LR5].ab1 Run Ended: 2026-07-03 06:23:55 Signal G:616 A:845 T:1364 C:1762

Sample: 72010503276_32026070202233_(533)_[LR5]_PW Lane: 4 Base spacing: 14.71 954 bases in 11457 scans

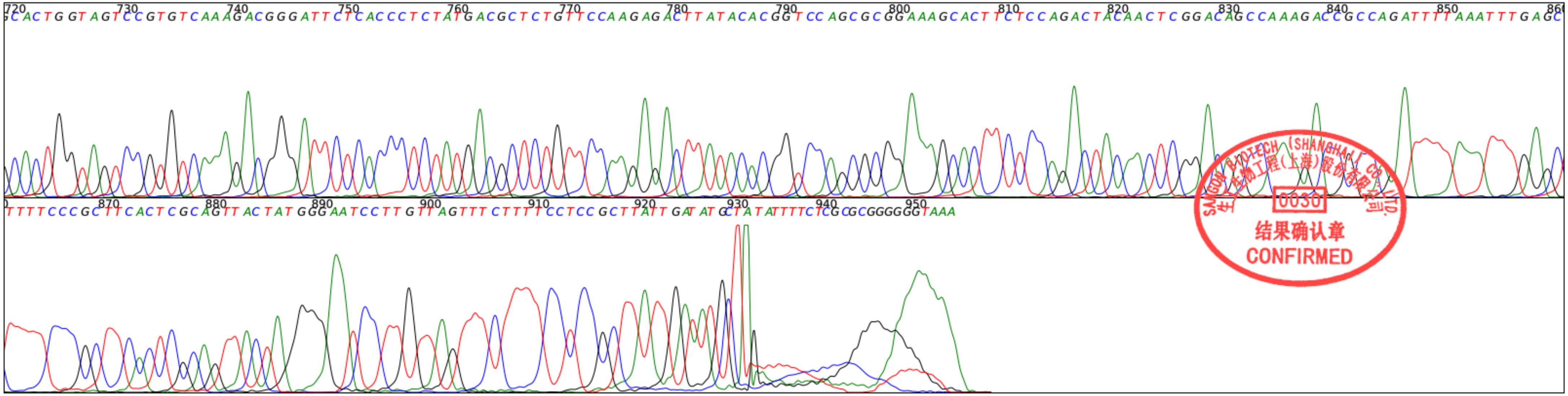

Supplement: Supplementary file 1 [file mycokeys-139-341-s001.zip › 序列/Type of Polyporus laoshanensis_QYZhang533/LSU/0014_32026070202233_(533)_[LR5]_H.pdf]
