# Supplementary material for: Two new polypore species Coriolopsis albescens and Polyporus laoshanensis (Basidiomycota, Polyporales) from Jiangsu Province, China
Source: MycoKeys. 2026 Sep 4;139:341–62. doi: 10.3897/mycokeys.139.209057 (PMC13570254; doi:10.3897/mycokeys.139.209057)

File: 0009_32025120503894_(533)_[ITS5].ab1 Run Ended: 2025-12-06 05:17:02 Signal G:1157 A:2352 T:4603 C:3327

Sample: 72009541198_32025120503894_(533)_[ITS5]_PW Lane: 4 Base spacing: 14.51 648 bases in 7789 scans

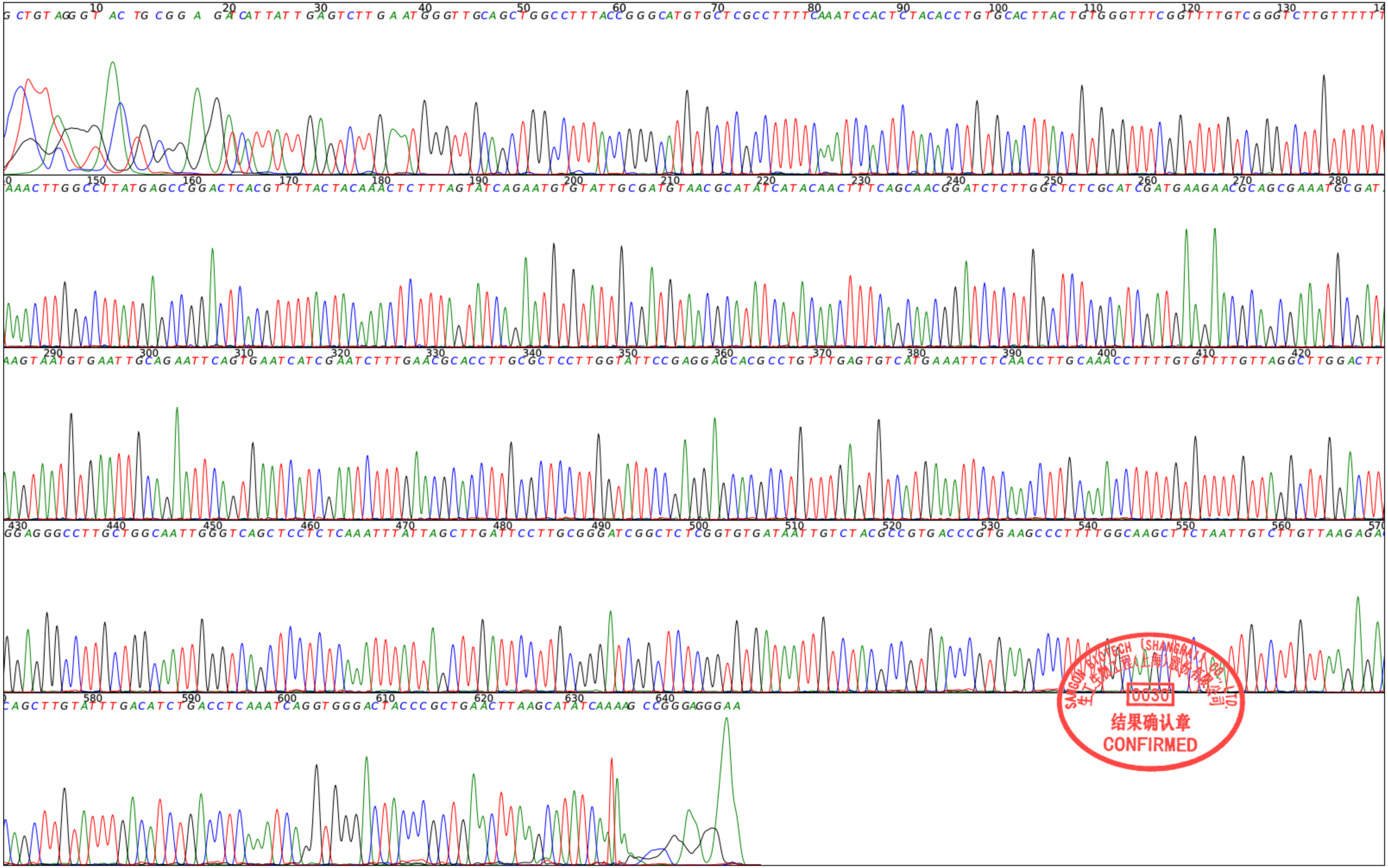

Supplement: Supplementary file 1 [file mycokeys-139-341-s001.zip › 序列/Type of Polyporus laoshanensis_QYZhang533/ITS/0009_32025120503894_(533)_[ITS5]_H.pdf]
